# Comparing downstream consequences of normal exercise stress echocardiograms and cardiac computed tomography angiography scans in patients suspected of having of obstructive coronary artery disease: a retrospective cohort study of Tricare beneficiaries

**DOI:** 10.1007/s10554-021-02343-8

**Published:** 2021-08-06

**Authors:** Cory G. Madigan, Michael B. Adams, Chu-Chiao Chu, Laith R. Dinkha, Samuel J. Farrell, Robert T. Hoard, Andrea N. Keithler, Kevin A. Loudermilk, Jessica Rouse, Brandon L. Walker, Susan G. Williams, Andrew C. Wyatt, Rosco S. Gore, Dustin M. Thomas

**Affiliations:** 1 Department of Cardiology, Brooke Army Medical Center, San Antonio, TX USA; 2 Parkview Health, Ft. Wayne, IN USA

**Keywords:** Cardiac CT, Stress echocardiography, Downstream testing, Coronary artery disease

## Abstract

To compare overall number of downstream tests and total costs between negative exercise stress echocardiograms (ESE) or cardiac computed tomography angiography scans (CCTA) in symptomatic Tricare beneficiaries suspected of having coronary artery disease (CAD). This is a retrospective cohort study examining 651 propensity-matched patients who underwent ESE or CCTA with normal results between 2008 and 2014 at the United States’ largest Department of Defense hospital. The total number of additional downstream tests over the next five years was determined. The total costs associated with each arm, inclusive of the initial test and all subsequent tests, were calculated using the 2018 Medicare Physician Fee Schedule. 18.5 percent of patients with a normal ESE result underwent some additional form of cardiac testing over the five years after initial testing compared to 12.8 percent of patients with a normal CCTA. The absolute difference in total number of downstream tests between both study groups was 5.7 percent (p = 0.03). When factoring the costs of the initial test as well as the downstream tests, the ESE group was associated with overall lower costs compared to the CCTA group, 351 United States Dollars (USD) versus 496 USD (p < 0.0001). This study demonstrates that, when compared to CCTA, ESE is associated with a higher total number of downstream tests, but overall lower total costs when chosen as initial testing strategy for suspected CAD.

## Introduction

Coronary artery disease (CAD) remains a leading cause of death around the world. The clinician evaluating the patient suspected of having symptoms attributable to stable obstructive CAD is confronted with the decision of choosing the most appropriate test. Exercise stress echocardiography (ESE) is a well-validated form of functional testing boasting a high sensitivity and specificity for the detection of obstructive CAD with the notable benefits of widespread availability and the absence of ionizing radiation exposure. Cardiac computed tomography angiography (CCTA) has established itself as a commonly utilized noninvasive alternative to invasive coronary angiography for anatomic evaluation of coronary anatomy. Multiple randomized controlled trials and subsequent meta-analyses have demonstrated that CCTA can reliably exclude obstructive CAD with a very high sensitivity and negative predictive value (NPV) [1–4]. Furthermore, two randomized controlled trials (RCTs), the Prospective Multicenter Imaging Study for Evaluation of Chest Pain (PROMISE) and Scottish Computed Tomography of the Heart (SCOT-HEART) trials, have confirmed the utility of an anatomic approach to CAD detection when compared to a functional stress-testing approach for outpatients with intermediate pre-test risk who present in the outpatient setting with symptoms concerning for obstructive CAD [5, 6]. However, few studies have directly compared ESE directly with CCTA. Of relevance to our study design, Hadamitzky et al. have shown that the event-free five-year survival is greater than 97% in patients without obstructive disease on CCTA [7]. In light of this, some authors have described a “warranty period” following a normal CCTA [8]. Given that many clinicians may feel more comfortable forgoing additional testing with normal CCTA results, our study was designed to specifically examine the downstream consequences of patients undergoing CCTA or ESE who have an initial negative test result.

## Methods

### Study design

This is a retrospective study of symptomatic patients without known CAD who underwent noninvasive testing for CAD between the January 1, 2008 and June 31, 2014 at Brooke Army Medical Center, the largest hospital in the Department of Defense. The aim of the study was to compare the downstream consequences between those with a negative test result when initial testing was ESE or CCTA. The patient population includes active-duty personnel, their family members, and retired military beneficiaries. The electronic medical record was queried to identify individuals who underwent cardiac stress testing during the study period. To be included in the study, patients required symptoms potentially attributable to myocardial ischemia (see Table 1), had to be between 45 and 70 years old at the time of initial testing, have a minimum presence of one cardiac risk factor and have a normal initial test result. Indications for testing were obtained by reviewing the patient’s electronic medical record. Normal CCTA was defined as Coronary Artery Disease Reporting and Data System (CAD-RADS) 2 or less. This equates to absence of any stenosis greater than 50%. Stress echocardiography was considered normal if the patient reached target heart rate and normal augmentation of ejection fraction and absence of any stress-induced wall motion abnormality. Electrocardiographic changes, blood pressure response, and other non-imaging portions of ESE were not included in our study. Exclusion criteria included low pretest cardiovascular risk defined as CAD Consortium Score of less than 5%, and an abnormal or non-diagnostic initial cardiac testing.

**Table 1:** Baseline characteristics prior to propensity matching

	CCTA (*n* = 1285)	Stress Echo (*n* = 327)	*p-value*
Male Sex, no. (%)	790 (61%)	180 (55%)	0.0347
Age (years)			
Mean ± SD	52 ± 8.9	53 ± 8.7	0.783
Range	35–70	35–70	
Hypertension, no. (%)	661 (51%)	180 (55%)	0.2487
Hyperlipidemia, no. (%)	606 (47%)	151 (46%)	0.7506
Diabetes mellitus, no. (%)	152 (12%)	63 (19%)	0.0007
Active smoker, no. (%)	150 (12%)	32 (10%)	0.3282
CAD consortium score (%)	6.1 ± 8.1	10.4 ± 11.1	< 0.0001
Indication, no. (%)			0.0996
ATCP	989 (77%)	235 (72%)	
Angina	29 (2%)	9 (3%)	
Dyspnea	45 (4%)	22 (7%)	
Palpitations	56 (4%)	15 (4%)	
Syncope	8 (1%)	5 (1%)	
Other	158 (12%)	41 (13%)	

Measures were taken to protect the privacy of the subjects. Those who met the inclusion criteria were assigned a unique study code which did not contain any personally identifiable information. The subject identifiers required for data collection were maintained separately from the study database in a secure, password-protected location. Upon completion of the study, the master file was deleted, rendering the data set de-identified.

Each subject’s gender, cardiac risk factors, study indication, study result, and downstream tests completed within five years of the initial evaluation were documented and analyzed. Downstream tests were defined specifically as additional tests evaluating for myocardial ischemia. Costs of downstream testing were determined using the Medicare Physician Fee Schedule 2018 final rule.

Data analysis was performed using the Statistical Analysis System (SAS) software, including two-sided Chi-squared testing of categorical variables, analysis of variance (ANOVA) of continuous variables with normal distribution, and Wilcoxon signed-rank test and the Kruskal–Wallis Test for non-normally distributed continuous variables. Univariate and multivariate regression were completed to determine factors that might lead to increased testing. Propensity matching using the CAD Consortium score was used to compare CCTA with ESE.

## Results

In the specified six-and-a-half-year time interval, a total of 11,636 patients undergoing any form of noninvasive cardiac testing were screened. Of those screened, 2,864 patients underwent CCTA and 1,285 of those were included after excluding patients who did not meet study parameters described above. A total of 985 patients who underwent ESE were screened. Of these, 327 were eligible for inclusion. The initial patient demographics are displayed in Table 1. There were statistically significant baseline differences in the populations of patients undergoing CCTA versus ESE. Notably, patients undergoing ESE were more likely to be female and have diabetes mellitus. After propensity matching, there remained 327 and 324 patients in the CCTA and ESE cohorts, respectively. The patients had similar average CAD consortium scores around 10%. The demographics of the patients included in each propensity-matched cohort are displayed in Table 2.

**Table 2:** Baseline characteristics after propensity matching

Characteristic	CCTA (*n* = 327)	Stress Echo (*n* = 324)	*p-value*
Male Sex, no. (%)	197 (60%)	177 (55%)	0.147
Age (years)			
Mean ± SD	51 ± 8.6	51 ± 8.7	0.659
Range	35–70	35–70	
Hypertension, no. (%)	160 (49%)	177 (55%)	0.146
Hyperlipidemia, no. (%)	153 (47%)	148 (46%)	0.776
Diabetes Mellitus, no. (%)	41 (13%)	61 (19%)	0.0269
Active Smoker, no. (%)	31 (9%)	31 (10%)	0.970
CAD Consortium Score (%)	10.9 ± 7.7	10.1 ± 7.2	0.1544
Indication, no. (%)			0.4916
ATCP	245 (75%)	233 (72%)	
Angina	9 (3%)	8 (2%)	
Dyspnea	18 (5.5%)	22 (7%)	
Palpitations	18 (5.5%)	15 (5%)	
Syncope	1 (0%)	5 (1%)	
Other	36 (11%)	41 (13%)	

According to the 2018 Final Medicare Physician Fee Schedule, the price of ESE was 239 United States Dollars (USD) whereas the cost of CCTA was 432 USD [9]. A total of 18.5 percent of patients with a normal ESE result underwent some additional form of cardiac testing in the next five years compared to 12.8 percent of patients with a normal CCTA. The absolute difference in total number of downstream tests between both study groups was 5.7 percent, correlating to an additional downstream test for every 17 ESE performed. When factoring the costs of the initial test as well as the downstream tests, the ESE group was associated with overall lower costs, 351 USD compared to the CCTA group which averaged 496 USD, p < 0.0001 (see Table 3).

**Table 3:** Differences in number and cost of downstream testing for propensity matched cohort of patients initially evaluated with CCTA and stress echocardiography

	CCTA (*n* = 327)	Stress Echo (*n* = 324)	*p-value*
Total cost of initial test (USD)	432.36	239.04	n/a
Number of downstream tests, no	42	60	0.0296
Percentage undergoing additional testing	12.8%	18.5%	0.0296
Overall testing cost (USD)	496.35 ± 185.35	351.48 ± 270.64	< 0.0001

## Discussion

This study set out to examine the downstream effects of negative ESE or CCTA when chosen as the initial noninvasive test for suspected CAD. As described in detail above, the choice of ESE was found to have a greater number of downstream tests, which is in line with the “warranty period” of CCTA. However, when accounting for the cost of the initial negative test, ESE was associated with a lower total cost. We originally hypothesized that CCTA would be associated with both total lower number of downstream tests and total cost due to the previously published negative predictive value of CCTA in RCTs.

In the past few years, two large RCTs, PROMISE and SCOT-HEART, have compared clinical outcomes between groups randomized to functional or anatomic testing with CCTA. As a result of these two and other studies, the European Society of Cardiology (ESC) guidelines for chronic coronary endorsed CCTA as a Class I recommendation for initial test to diagnose CAD [10]. As found in the PROMISE trial, there was no major difference between the CCTA and functional testing arms when comparing the primary composite outcome of death from any cause, nonfatal myocardial infarction (MI), hospitalization for unstable angina, or procedural complication. However, the CCTA arm showed statistically significant increases in total radiation, a 4.1% absolute increase in diagnostic cardiac catheterizations, and a 3.0% absolute increase in revascularization [6]. The SCOT-HEART trial remarkably showed that patients undergoing an anatomic evaluation with CCTA had a lower primary endpoint of death from CAD or nonfatal MI than the standard care group, predominately driven by nonfatal MI [11]. The authors hypothesized that this was due to increased preventive therapy for primary prevention of MI including statins, aspirin, lifestyle interventions, and revascularization when appropriate. They also hypothesized this may be due to increased patient motivation given objective measure of disease.

Notably, ESE was underrepresented in the functional imaging control group in both landmark studies. Specifically, only 22% of patients underwent ESE compared to 67% undergoing nuclear testing in the PROMISE trial [6]. Less than one percent of patients underwent ESE in the SCOT-HEART trial [5]. Furthermore, while the authors are aware of at least one prospective RCT comparing clinical outcomes between myocardial perfusion imaging (MPI) and CCTA, we are unaware of any studies directly comparing ESE to CCTA in the outpatient setting [12]. Upon our review of the literature, there has only been one RCT comparing ESE vs CCTA. Levsky et al. enrolled 400 patients without known CAD presenting with chest pain to the emergency department and showed that ESE and CCTA led to similar results in major adverse cardiac events (MACE), invasive angiography, and revascularization by one year. Patients in the CCTA arm were admitted more often and spent more days in the hospital than the patients in the ESE group [13].

Moreover, ESE possesses a few clear advantages when compared to CCTA. Despite the growing use of CCTA, ESE remains widely available in the clinic and emergency department settings with minimal equipment requirements. In addition to wall motion analysis, additional prognostic data can be derived, such as metabolic equivalents, heart rate response, and exercise induced hypertension. For patients being referred for cardiac testing with chief complaint of dyspnea, ESE can provide diagnostic information in nonischemic etiologies, including exercise-induced diastolic dysfunction, pulmonary hypertension, and severity of mitral valve disease. Importantly, ESE does not expose the patient to ionizing radiation. These advantages are in addition to the post cost savings discussed above.

Our study has several strengths. First, as fewer downstream tests were performed in the CCTA arm, it shows that providers likely act in light of the perceived “warranty period” of a normal CCTA. Additionally, our study benefits from the comprehensive electronic medical record available through the Department of Defense health system. As a result, over 11,000 patients were screened. Lastly, propensity matching was completed as outlined above, further strengthening the comparison between the two arms [4].

**Fig. 1:**
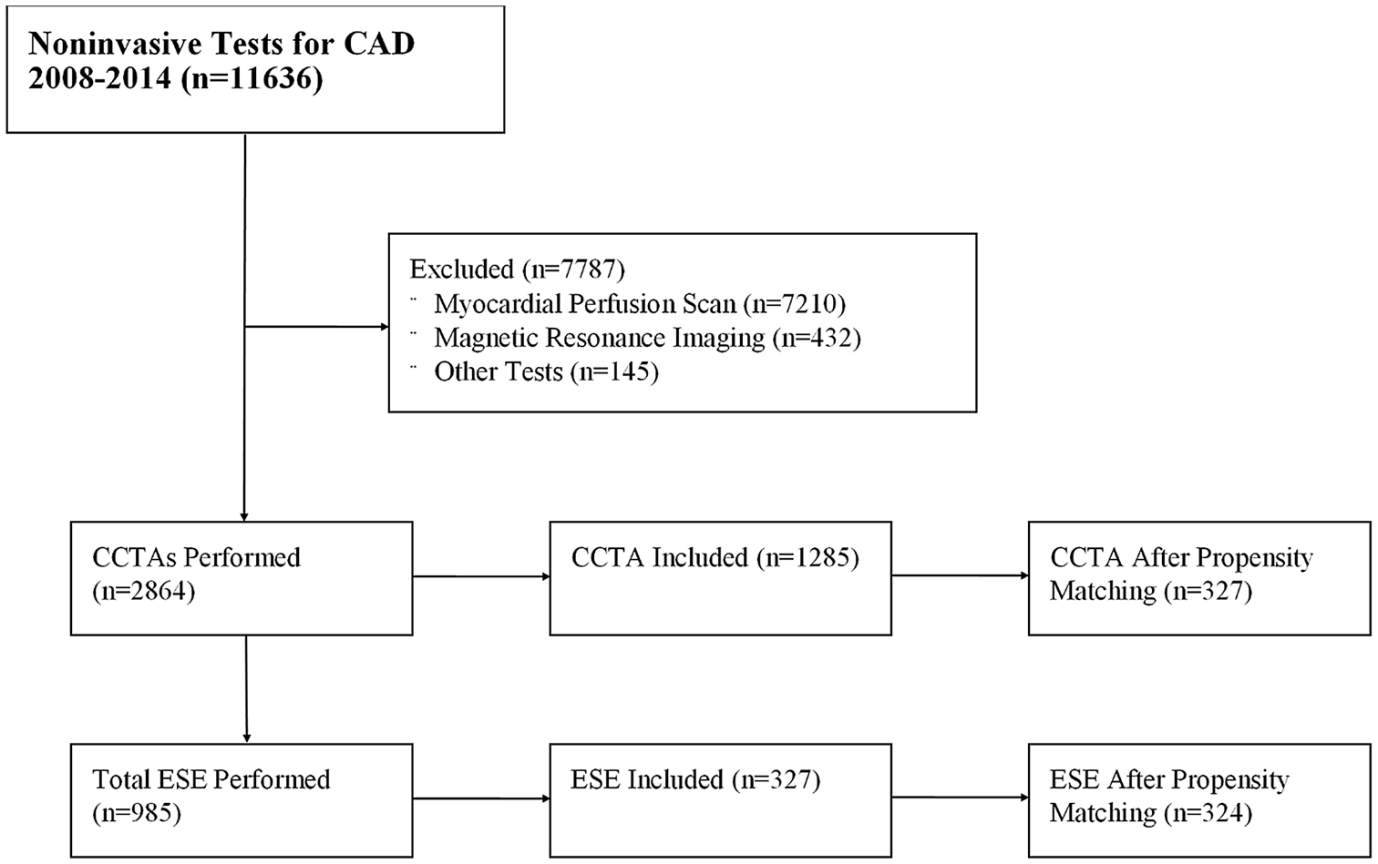
Screening, inclusion, and propensity matching. *CCTA* coronary computed tomography angiography. *ESE* exercise stress echocardiogram. Figure created in Microsoft Publisher

Our trial has some limitations. Most importantly, it should be noted that the cost-analysis cannot be used to compare CCTA and ESE as a whole because our analysis does not include patients with positive results. Further, while the studied populations underwent propensity matching, this cannot eliminate all potential cofounders present in this retrospective analysis. Second, the results may lack generalizability given the population studied was Tricare beneficiaries. This is exemplified by the notable low pre-test probability noted in our study even after excluding very-low risk patients with a CAD consortium pretest probability of less than five percent. Last, it is retrospective and thus it should principally be viewed as hypothesis-generating (Fig. 1).

This study demonstrates an association between ESE with higher total number of downstream tests as well as lower costs when compared to CCTA. These findings are unexpected and potentially worthwhile as the cardiology community moves towards CCTA as a first line test for suspected CAD. As these findings are retrospective, future RCTs specifically examining the financial and clinical outcomes of CCTA compared with ESE would bring further clarity to these questions.

## Data Availability

This data was obtained through the electronic medical record and is not publicly available.

## Code availability

Statistical Analysis System was used for statistical analysis.
